# Association between pattern of hepatic iron deposition and severity of MASH among patients enrolled in the NASH Clinical Research Network

**DOI:** 10.1097/HC9.0000000000000847

**Published:** 2025-11-20

**Authors:** Aalam Sohal, Laura A. Wilson, David E. Kleiner, Rohit Loomba, Liyun Yuan, Srinivasan Dasarathy, Arun J. Sanyal, Brent A. Neuschwander-Tetri, Naga P. Chalasani, Anna Mae Diehl, Kris V. Kowdley

**Affiliations:** 1 Liver Institute Northwest, Seattle, Washington, USA; 2 Department of Epidemiology, Bloomberg School of Public Health, Johns Hopkins University, Baltimore, MD; 3 Laboratory of Pathology, National Cancer Institute, Bethesda, MD; 4 Division of Gastroenterology and Hepatology, University of California at San Diego, La Jolla; 5 Division of Gastroenterology and Hepatology, University of Southern California, Los Angeles, California, USA; 6 Division of Gastroenterology and Hepatology, Cleveland Clinic, Cleveland, Ohio, USA; 7 Division of Gastroenterology and Hepatology, Virginia Commonwealth University, Richmond, Virginia, USA; 8 Division of Gastroenterology and Hepatology, Saint Louis University School of Medicine, St. Louis, MO; 9 Division of Gastroenterology and Hepatology, Indiana University School of Medicine, Indianapolis, Indiana, USA; 10 Division of Gastroenterology and Hepatology, Duke University, Durham, North Carolina, USA

**Keywords:** biopsy, hepatocellular, iron deposition, MASH, NASH CRN, reticuloendothelial

## Abstract

**Background:**

We previously showed that the pattern of liver biopsy iron staining is associated with metabolic dysfunction–associated steatohepatitis (MASH) severity and fibrosis. The goal of the current study was to further examine the relationships between hepatic iron deposition and disease severity in a large biopsy-proven cohort of nonalcoholic Steatohepatitis Clinical Research Network (NASH CRN).

**Methods:**

Adult patients enrolled in the NASH CRN Non-alcoholic Fatty Liver Disease (NAFLD) Database, PIVENS, or FLINT studies from November 2004 to May 2022 were included. Patient demographics, clinical, and laboratory data were compared between patients with different iron deposition patterns. Multivariate logistic regression analysis was performed to identify the relationship between the iron deposition pattern and advanced fibrosis.

**Results:**

A total of 2833 patients were included; 1166 patients (41%) had positive hepatic iron staining. Two hundred seventy-two (9.6%) patients had hepatocellular (HC) iron, 284 (10%) had iron in the reticuloendothelial system (RES), and the remaining (21.4%) had mixed iron deposition. A higher proportion of patients with RES iron had grade 3 steatosis (29%), severe hepatocyte ballooning (47%), severe lobular inflammation (15%), cirrhosis (16%), and definite MASH (71%). On multivariate regression analysis, patients with RES iron had higher odds of advanced fibrosis (adjusted OR: 1.34, 95% CI: 1.11–1.62, *p*=0.003), while patients with HC iron had lower odds of advanced fibrosis (aOR: 0.68, 95% CI: 0.55–0.83, *p*<0.001).

**Conclusions:**

Hepatic iron is common in patients with MASH. Patients with a RES iron pattern have a severe disease, while those with HC iron have milder disease. These data provide insights into the potential role of hepatic iron in the pathogenesis of MASH.

## Graphical abstract

**Figure:**
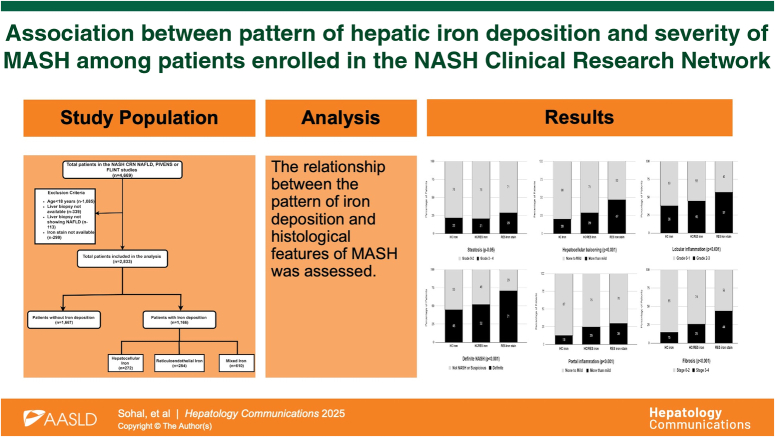

## INTRODUCTION

Metabolic dysfunction–associated steatotic liver disease (MASLD), previously called NAFLD, is the most common cause of liver disease and has been reported to affect about 30% of the global population.^1^,^2^ Patients with MASLD have an increased risk of cardiovascular events.^3^ However, in about 20% of patients with MASLD, the disease can progress to metabolic dysfunction–associated steatohepatitis (MASH), with its sequelae being advanced fibrosis and cirrhosis.^4^ Among these patients, the risk of developing liver-related adverse events is significantly higher.^5^

An active area of research in the last several decades has been into the various mechanisms that drive the progression of MASLD to MASH and fibrosis. It was initially suggested by Letteron et al^6^ that the mere presence of oxidizable fat could lead to lipid peroxidation. However, subsequent studies reported that a second hit in the form of oxidative stress was needed for progression to necroinflammation and fibrosis.^7^,^8^ Iron is critical for body functions, although, in its free form, it can generate toxic free radicals and reactive oxygen species, which may exacerbate liver injury and promote fibrogenesis.^9^ The prevalence of hepatic iron deposition among patients with MASLD has been reported to range from 35% to 48%.^10^,^11^ Due to its high prevalence and potential to cause oxidative stress, there has been interest in understanding the role of iron and HFE mutations in the pathophysiology and progression of the disease.

Studies have aimed to assess the association between *HFE* mutations and the risk of MASLD.^12^,^13^ A recent study by Sun et al^12^ reported that *HFE* plays a potential role in the development of steatosis among patients with lean MASLD. In a meta-analysis performed in 2013, Caucasians with *HFE* gene mutations were not noted to have an increased risk of MASLD.^14^ There are also conflicting data regarding the role of *HFE* mutations in the progression and severity of MASH.^15^,^16^ Our initial study of 126 patients reported that the presence of the *HFE C282Y* mutation was associated with advanced hepatic fibrosis among Caucasians with MASH.^15^ On the contrary, Chitturi et al^16^ failed to demonstrate an association between *HFE* mutations and advanced fibrosis among patients with MASLD. Bugianesi et al^17^ and Yoneda et al^18^ also reported no association between the mutations and disease course. Our subsequent follow-up study, using a larger cohort of 786 adult subjects, also failed to demonstrate an association between *HFE* mutations and advanced fibrosis.^19^ However, the study found that patients with *HFE* mutations had increased hepatocellular iron and lower hepcidin levels but no increased reticuloendothelial system (RES) deposition.^19^

Iron deposition in patients with MASH has been reported to be a significant predictor of advanced fibrosis.^20^ Subsequent studies reported that not only the iron concentration but the pattern of iron deposition may also play a role in the progression of chronic liver diseases.^10^,^11^,^21^ Three patterns of hepatic iron deposition have been described in the literature: hepatocelullar (HC), RES, and a mixed pattern.^10^,^11^ In 2010, Valenti et al^11^ analyzed 587 Italian patients with MASLD and reported that the presence of hepatocellular iron was associated with a higher likelihood of fibrosis above stage 1 among these patients. In 2011, our group performed a study on 849 subjects enrolled in the NASH CRN and reported that the presence of iron in the RES compartment was associated with advanced fibrosis, whereas the presence of HC iron was not.^10^ Since then, multiple studies have provided mechanistic insights regarding the role of RES iron in the progression of the disease.^22^,^23^,^24^ Our current study aimed to expand the existing literature by examining a cohort of 2833 patients enrolled in the NASH Clinical Research Network (NASH CRN) database with biopsy-proven MASLD and systematic assessment of hepatic iron deposition.

## METHODS

### Study population

**FIGURE 1:**
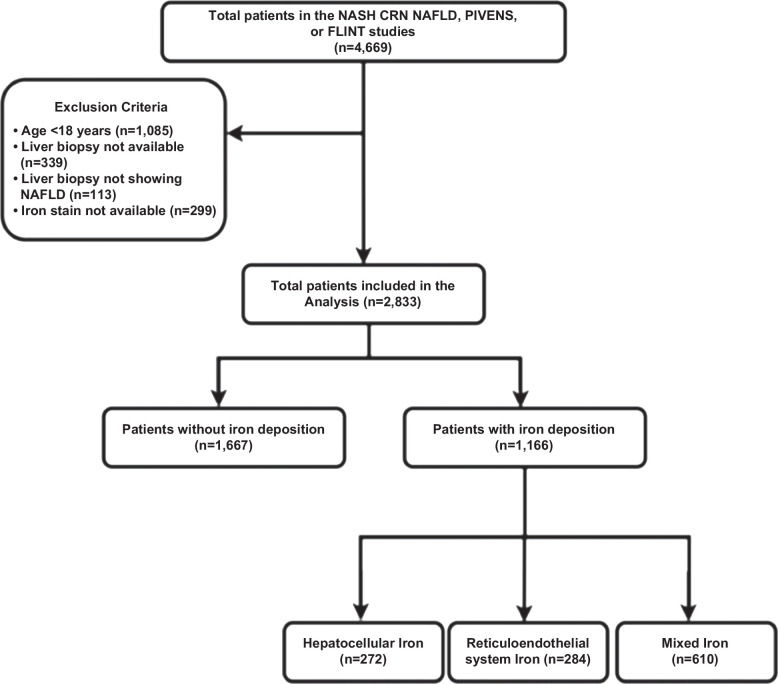
Inclusion flow diagram for the study population. Abbreviation: NASH CRN, nonalcoholic Steatohepatitis Clinical Research Network.

Participants enrolled in the NASH CRN NAFLD Database, PIVENS, or FLINT studies from November 2004 to May 2022 were included in the analysis. The study was conducted in accordance with both the Declarations of Helsinki and Istanbul. All clinical sites participating in the trial obtained approval from the institutional review board, and all patients were provided with written informed consent before enrollment in the study. The inclusion criteria for the database studies have been presented elsewhere.^22^,^23^ NASH CRN also excludes patients with known hemochromatosis (Hepatic Iron Index ≥1.9 or removal of >4 g of iron by phlebotomy), C282Y homozygosity for the *HFE* gene, or unexplained hepatic iron overload. The PIVENS and FLINT trials also excluded patients with other chronic liver diseases, including hemochromatosis. Out of the total of 4669 patients, 2833 patients were included in the analysis. The exclusion and inclusion criteria are summarized in Figure 1.

### Study variables

Information was collected regarding the patients’ demographics, such as age, sex, ethnicity, and race. Data on body weight and height were collected from physical exams performed on all subjects closest to the biopsy. In addition, laboratory data obtained within 6 months of the liver biopsy were compared between iron stain positive and negative subjects, if available (n=2473).

### Histological analysis

The Pathology Committee of the NASH CRN assessed the histological features of steatotic liver disease and iron staining patterns in a centralized consensus review format using criteria that have been described previously.^25^ The pathology committee was blinded to all clinical, laboratory, and demographic information. Iron staining was performed in a central lab using Perl’s iron stain. Iron stains were scored prospectively using a method agreed upon by the committee. Only granular iron deposition was scored, based on the agreement that only discernible granules represent significant iron deposition. Hepatocellular iron was scored from 0 to 4 using the method of Rowe et al,^26^ with the modification that a ×20 objective was used instead of a ×25 objective. Nonhepatocellular iron (RES) was scored on a 3-point scale as none, mild, and more than mild.

### Statistical analysis

Categorical variables are reported as N (%), while continuous variables were recorded as mean ± SD or median (IQR). Categorical variables, including histological features like steatosis grade and location, fibrosis stage, and lobular inflammation grade, were analyzed using the Fisher exact or chi-square tests. Continuous variables, such as laboratory measures, were not normally distributed and were analyzed using the Wilcoxon rank-sum or Kruskal-Wallis tests.

Multiple logistic regression analysis was performed to study the relationships between advanced fibrosis and the presence and grade of hepatocellular and RES iron. There were no patients with grade 4 hepatocellular iron; therefore, logistic regression analysis included patients up to grade 3 hepatocellular iron. A multivariate logistic regression model was adjusted for age at biopsy, sex, diabetes status, and BMI. All analyses were performed using STATA (version 17). Nominal, 2-sided *p* values were used and considered statistically significant if *p*≤0.05; no adjustments for multiple comparisons were made.

## RESULTS

### Characteristics

Out of 2833 patients included in the analysis, 1166 (41%) had positive hepatic iron staining on liver biopsy. A comparison of the entire cohort to patients with and without positive iron staining is presented in Table 1. There was a statistically significant sex difference, with a higher proportion of males in the iron stain group compared with those without stainable iron. Patients with stainable iron were slightly older (51.3 vs. 49.9 y). Iron staining was more common in non-Hispanics than in Hispanics.

**TABLE 1:** Comparison of patient characteristics, clinical, and laboratory values for subjects with different hepatic iron phenotypes

Characteristic	Iron stain negative (N=1667)	HC iron only (N=272)	RES iron only (N=284)	Mixed HC/RES Iron (N=610)	*p*
Male sex, n (%)	408 (24)	135 (50)	123 (43)	389 (64)	**<0.001**
Race, n (%)†					**<0.001**
White	1377 (86)	204 (77)	228 (85)	470 (79)	
Black	55 (3)	11 (4)	11 (4)	20 (3)	
Asian	70 (4)	30 (11)	22 (8)	76 (13)	
Other	94 (6)	19 (7)	8 (3)	32 (5)	
Hispanic, n (%)	228 (14)	30 (11)	29 (10)	44 (7)	**<0.001**
BMI (kg/m^2^)	35.2 (6.5)	32.6 (6.6)	35.0 (6.7)	32.8 (5.8)	**<0.001**
ALT (U/L)	56 (36, 85)	53 (33, 78)	69 (43, 111)	58 (39, 87)	**<0.001**
AST (U/L)	43 (30, 64)	35 (27, 49)	49 (31, 74)	39 (29, 54)	**<0.001**
AST/ALT	0.8 (0.6, 1.0)	0.7 (0.6, 0.9)	0.7 (0.6, 0.9)	0.7 (0.5, 0.8)	**<0.001**
Total bilirubin (mg/dL)	0.5 (0.4, 0.8)	0.6 (0.5, 0.9)	0.6 (0.5, 0.9)	0.7 (0.5, 0.9)	**<0.001**
HDL (mg/dL)	42 (36, 51)	43 (37, 51)	41 (33, 49)	41.0 (36, 49)	**0.04**
Insulin (µU/mL)	20.1 (13.0, 31.6)	17.0 (12.2, 26.6)	23.1 (14.8, 34.0)	17.7 (11.2, 26.1)	**<0.001**
HOMA-IR	5.2 (3.1, 9.3)	4.4 (2.8, 6.8)	5.9 (3.7, 10.7)	4.4 (2.6, 6.9)	**<0.001**
Platelets (K/mm^3^)	246 (203, 292)	237 (198, 278)	219 (173, 269)	210 (174, 255)	**<0.001**
Hemoglobin (g/dL)	13.8 (12.9, 14.7)	14.7 (13.8, 15.5)	14.5 (13.3, 15.2)	14.9 (13.9, 15.7)	**<0.001**
Serum iron (µg/dL)	78 (59, 100)	91 (73, 112)	83 (64, 100)	102 (80, 124)	**<0.001**
TIBC (µg/dL)	382 (342, 424)	351 (317, 387)	352.5 (319, 395)	332 (298, 369)	**<0.001**
Ferritin (ng/mL)	91.5 (49, 164)	160 (98, 235)	224 (129.5, 375)	335 (207, 530)	**<0.001**
TS (iron/TIBC)	0.21 (0.15, 0.27)	0.27 (0.20, 0.35)	0.23 (0.18, 0.30)	0.30 (0.23, 0.38)	**<0.001**

Bold signifies statistical significance.

* Chi-square for categorical measures, Kruskal-Wallis test for continuous measures presented as medians (IQR), and *t* test for BMI.

† N=106 participants declined to identify race.

Abbreviations: BMI, Body Mass Index; HC, hepatocellular; HOMA-IR, Homeostatic Model Assessment of Insulin Resistance; RES, reticuloendothelial system; TIBC, Total Iron Binding Capacity; TS, Transferrin Saturation.

### Laboratory data in subjects with and without stainable hepatic iron

Subjects with positive iron stains had higher ALT levels (*p*=0.02), lower platelet count (*p*<0.001), and higher total bilirubin (*p*<0.001). Markers of insulin resistance, such as insulin level and HOMA-IR, were higher in patients without stainable iron. No significant difference was noted in total cholesterol, LDL cholesterol, triglycerides, and glucose levels. Patients with stainable hepatic iron had higher serum iron, serum ferritin, and transferrin saturation (*p*<0.001). These patients were also noted to have lower serum Total Iron Binding Capacity levels (*p*<0.001). Results of laboratory tests among subjects with and without stainable hepatic iron are shown in Table 2.

**TABLE 2:** Comparison of the grading of iron among patients with various iron deposition patterns

Characteristic	Iron stain negative (N=1667)	HC iron only (N=272)	RES iron only (N=284)	Mixed HC/RES Iron (N=610)	*p*
HC iron grade, n (%)					**<0.001**
Absent of barely discernible (×40)	1667 (100)	0 (0)	0 (0)	0 (0)	
Barely discernible granules (x20)	0 (0)	238 (88)	0 (0)	393 (64)	
Discrete granules resolved (×10)	0 (0)	31 (11)	0 (0)	187 (31)	
Discrete granules resolved (×4)	0 (0)	3 (1)	0 (0)	30 (5)	
RES iron grade, n (%)					**<0.001**
None	1667 (100)	272 (100)	0 (0)	0 (0)	
Mild	0 (0)	0 (0)	241 (85)	390 (64)	
More than mild	0 (0)	0 (0)	43 (15)	220 (36)	

Bold signifies statistical significance.

Abbreviations: HC, hepatocellular; RES, reticuloendothelial system.

### Relationship between patterns of hepatic iron staining and clinical and laboratory differences

Three distinct patterns of hepatic iron staining were observed as follows:

1. Iron was localized to HC in 272/1166 subject biopsies (23.32%).
2. Iron was localized to the RES in 284/1166 subject biopsies (24.35%).
3. Iron in both HC and RES systems in 610/1166 subject biopsies (52.31%).

Subjects with RES iron had higher BMI compared with the other groups. Patients with RES iron deposition were also noted to have the highest AST, ALT, insulin, and HOMA-IR levels. Patients with mixed iron deposition had the lowest platelet count, followed by the RES iron group. Compared with other groups, the highest serum ferritin, iron, and transferrin saturation were noted in the patients with mixed iron deposition. Patients with mixed iron deposition also had the lowest total iron binding capacity. Geographical distribution by iron positivity is presented in Supplemental Table S1, http://links.lww.com/HC9/C200.

### Relationship between hepatic iron staining and histological severity

**FIGURE 2:**
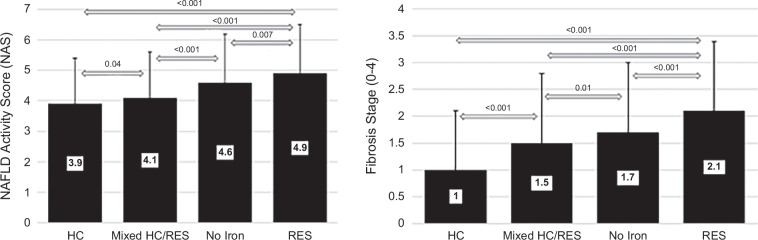
Comparison of mean NAS and fibrosis score among MASLD subjects with different iron staining patterns. SDs are indicated by error bars. Abbreviations: HC, hepatocellular; MASLD, metabolic dysfunction–associated steatotic liver disease; NAS, NAFLD activity score; RES, reticuloendothelial system.

There were significant differences across all groups in the proportion of subjects in different severity categories for portal inflammation, lobular inflammation, hepatocyte ballooning, and fibrosis. No significant difference was noted in the grades of steatosis across groups. Information regarding the histological grade and stage among various hepatic iron staining groups is presented in Table 3. The NAFLD activity score, as well as the fibrosis stage, was significantly different across all groups. The highest NAFLD activity score and fibrosis stage were noted in the RES group, while the lowest were in the HC group. The mean NAFLD activity score and fibrosis stage (range: 0–4) are presented in Figure 2.

**TABLE 3:** Histologic differences between iron-staining groups

Characteristic	Iron stain negative (N=1667)	HC iron only (N=272)	RES iron only (N=284)	Mixed HC/RES iron (N=610)	*p*
Steatosis grade, n (%)					0.06
5%–33%	666 (40)	118 (43)	109 (38)	273 (45)	
34%–66%	548 (33)	93 (34)	94 (33)	208 (34)	
>66%	453 (27)	61 (22)	81 (29)	129 (21)	
Fibrosis stage, n (%)					**<0.001**
None	370 (22)	106 (39)	39 (14)	164 (27)	
Mild to moderate, zone 3, perisinusoidal, or portal/periportal only	459 (28)	89 (33)	60 (21)	164 (27)	
1a	200 (12)	43 (16)	30 (11)	65 (11)	
1b	204 (12)	35 (13)	22 (8)	75 (12)	
1c	55 (3)	11 (4)	8 (3)	24 (4)	
Zone 3 and periportal, any combination	318 (19)	35 (13)	59 (21)	123 (20)	
Bridging	369 (22)	32 (12)	80 (28)	109 (18)	
Cirrhosis	150 (9)	10 (4)	46 (16)	50 (8)	
Lobular inflammation, n (%)					**<0.001**
0	8 (<1)	4 (1)	0 (0)	4 (1)	
<2 (×20)	850 (51)	166 (61)	121 (43)	333 (55)	
2-4 (×20)	615 (37)	91 (33)	119 (42)	227 (37)	
>4 (×20)	194 (12)	11 (4)	44 (15)	46 (8)	
Chronic portal inflammation, n (%)					**<0.001**
None	200 (12)	50 (18)	25 (9)	82 (13)	
Mild	1056 (63)	188 (69)	173 (61)	377 (62)	
More than mild	411 (25)	34 (13)	86 (30)	151 (25)	
HC ballooning, n (%)					**<0.001**
None	528 (32)	134 (49)	72 (25)	254 (42)	
Few	454 (27)	84 (31)	79 (28)	181 (30)	
Many	685 (41)	54 (20)	133 (47)	175 (29)	
MASH diagnosis, n (%)					**<0.001**
Not MASH	324 (19)	96 (35)	41 (14)	162 (27)	
Suspicious/borderline	323 (19)	54 (20)	42 (15)	129 (21)	
Definite	1020 (61)	122 (45)	201 (71)	319 (52)	

Bold signifies statistical significance.

* Chi-square test was performed.

Abbreviations: HC, hepatocytes; MASH, metabolic dysfunction–associated steatohepatitis; RES, reticuloendothelial system.

**FIGURE 3:**
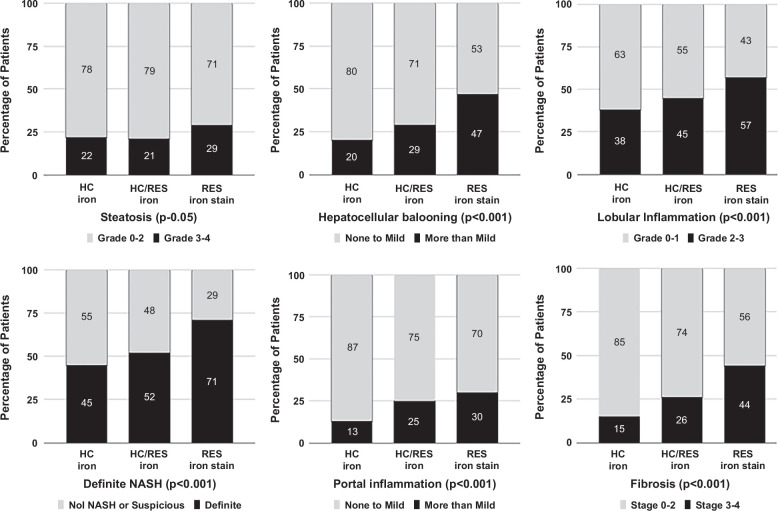
Relationship between histologic features and iron staining pattern among subjects with stainable iron. Abbreviations: HC, hepatocellular; RES, reticuloendothelial system.

Patients with the RES pattern had the highest proportion of patients in the most severe category for each histological feature. Patients with HC patterns had the lowest proportion in the least severe category for each histological feature, except for steatosis. The differences in the proportion of subjects with advanced histological features were statistically significant across different iron groups (*p*<0.001 in all groups except steatosis, *p*=0.06). This information is presented in Figure 3.

### Results of multiple logistic regression analysis identifying an association between pattern/grade of iron deposition and advanced fibrosis and MASH

**FIGURE 4:**
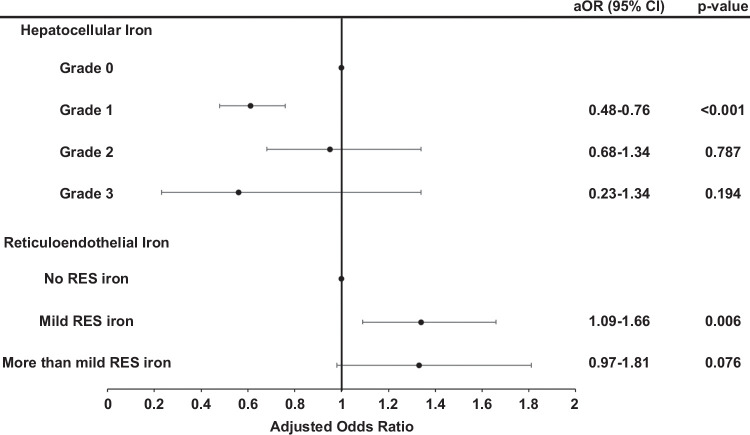
Multiple logistic regression analysis used to model the independent risk for the grade of HC and REC iron on the occurrence of advanced fibrosis (yes vs. no). Each predictor in the table was modeled individually after adjustment for age at biopsy, sex, diabetes, and BMI. Abbreviations: BMI, Body Mass Index; HC, hepatocytes; RES, reticuloendothelial system.

On multiple logistic regression analysis, the presence (OR: 1.34, 95% CI: 1.11–1.62, *p*=0.003) but not the grade of RES iron (OR: 1.2, 95% CI: 0.89–1.62, *p*=0.825) was positively associated with advanced fibrosis after adjusting for age at biopsy, sex, diabetes status, and BMI. On assessing the grade of RES iron deposition, mild RES iron deposition was associated with higher odds of advanced fibrosis (aOR: 1.34, 95% CI: 1.09–1.66, *p*=0.006). A trend toward higher odds of advanced fibrosis was noted in patients with more than mild advanced fibrosis (aOR: 1.33, 95% CI: 0.97–1.81), although not statistically significant. On the contrary, patients with HC iron pattern had a lower risk of advanced histological features of MASH (aOR: 0.68, 95% CI: 0.55–0.83, *p*<0.001). A higher grade of hepatocellular iron deposition had no association with advanced fibrosis (aOR: 1.03, 95% CI: 0.76–1.42, *p*=0.232). On assessing the grade of HC iron deposition, grade 1 was associated with lower odds of advanced fibrosis (aOR: 0.68). However, no significant difference was noted between grade 2, grade 3, and hepatocellular iron. The results are presented in Table 4 and Figure 4.

**TABLE 4:** Results of multiple logistic regression analysis used to model the independent risk for the presence and grade of HC and REC iron on the occurrence of advanced fibrosis (yes vs. no)

	Adjusted OR (lower 95% CI)	*p*
Hepatocellular iron	0.68 (0.55–0.83)	**<0.001**
Grade 1 vs. Grade 0	0.61 (0.48–0.76)	**<0.001**
Grade 2 vs. Grade 0	0.95 (0.68–1.34)	0.787
Grade 3 vs. Grade 0	0.56 (0.23–1.34)	0.194
Reticuloendothelial iron	1.34 (1.11–1.62)	**0.003**
Mild iron deposition vs. none	1.34 (1.09–1.66)	**0.006**
More than mild iron deposition vs. none	1.33 (0.97–1.81)	0.076

Each predictor in the table was modeled individually after adjustment for age at biopsy, sex, diabetes, and BMI. Bold signifies statistical significance.

Abbreviations: BMI, Body Mass Index; HC, hepatocellular; RES, reticuloendothelial system.

## DISCUSSION

The current study is the largest study of patients with MASLD examining the pattern of iron deposition, severity of MASH, and fibrosis stage in a well-characterized cohort. The presence of RES iron was associated with higher severity of MASH and increased odds of advanced fibrosis. In contrast, HC iron was associated with lower odds of advanced fibrosis, a novel finding not noted in our previous study.^19^ The presence of HC iron was also associated with milder MASLD compared with those without stainable iron on liver biopsy. These findings indicate a possible role of RES iron as a cofactor in disease progression.

Several recent scientific observations have been made that support the role of RES iron as a cofactor in the progression of MASH. In 2013, Maliken and colleagues analyzed 83 patients with biopsy-proven MASLD. They reported the presence of RES iron to be associated with increased markers of oxidative stress, apoptosis, and MASH, while the presence of HC iron was only associated with increased oxidative stress but not apoptosis.^22^ In our current study, patients with MASH and HC iron had lower serum insulin and HOMA-IR levels. We hypothesize that the presence of HC iron among these patients might have led to the development of MASH among these patients due to increased oxidative stress. This is in agreement with the previous study by Handa et al^24^ that reported chronic dietary iron overload, even in the absence of an obesogenic diet among mice, resulted in steatohepatitis. Handa et al^24^ also reported patients with RES iron to have higher expression of macrophage M1 proinflammatory markers and reduced M2 anti-inflammatory markers compared with those with HC iron deposition. A recent paper by Gao et al^27^ provided important mechanistic insights regarding the role of iron among patients with MASH. Their study using mouse models reported that total iron levels in hepatocytes of patients with MASH were lower compared with healthy hepatocytes.^27^ They hypothesized that iron deficiency in hepatocytes might contribute to hepatic lipogenesis and that the presence of iron precedes steatosis.^27^ Gao et al^27^ proposed that hepatocyte iron deficiency is mediated by extracellular vesicles that transport HC iron to KCs and lead to an increase in HSC iron overload.

Since our original report of the relationship between hepatic iron deposition and MASH severity, ferroptosis has been described as a distinct iron-dependent cell death pathway. Ferroptosis has been implicated in triggering the progression of MASLD to MASH and fibrosis.^28^ Tsurusaki et al^29^ reported ferroptosis to precede cell apoptosis and thus accelerate the progression of MASH. Qi et al^30^ reported ferroptosis to exacerbate inflammatory response and oxidative stress during the early stages of MASH. Ferroptosis has been shown to cause pancreatic beta-cell loss and thus increase insulin resistance, a driving factor for the development of MASH.^31^ It has also been suggested that ferroptosis inhibition may prevent the development of MASH. Further research examining ferroptosis inhibition as a potential target for MASH is needed.

We previously suggested the mechanism by which tonic or phasic alterations in circulating hepcidin levels might contribute to iron accumulation in the HC and RES compartments.^32^ Hepcidin is a key regulator of iron homeostasis and is regulated by both total body iron stores as well as inflammation.^32^ It has been suggested that patients with MASH and MASLD have increased hepatic hepcidin levels. We previously reported that patients with mixed HC/RES iron had high serum levels of hepcidin, while patients with HC iron had lower serum hepcidin levels than patients with RES iron.^19^ Increased serum hepcidin levels would contribute to increased iron sequestration in the macrophages. Since hepcidin is also regulated by inflammation, it is likely that higher levels of serum hepcidin in patients with more severe MASH may lead to increased iron sequestration in the hepatocytes. By contrast, we believe the hepatocyte pattern of iron deposition is likely due to mutations in genes regulating iron homeostasis. Our group has previously reported that about 40% of the patients with MASH have common mutations in the *HFE* gene.^19^ HC iron loading among these patients is likely a consequence of reduced hepcidin levels secondary to these mutations.^32^ Another finding that supports the pathophysiology of HC iron deposition is by Hoki et al,^33^ who, in their study of 40 Japanese patients with MASLD/MASH, reported that patients with MASH have upregulation of divalent metal transporters, even in the presence of elevated hepcidin.

It remains unclear whether iron accumulation in RES is the cause or result of severe MASH. An alternate explanation for RES iron accumulation is that it is a byproduct of increased apoptosis rather than the cause. To answer this question definitively, longitudinal studies of disease progression with histologic and clinical data of patients with various patterns of iron deposition are needed. However, the current evidence is compelling that the presence of RES iron is a driving factor for increased inflammation and fibrogenesis among patients with MASLD. An intriguing observation in 299 patients in Austria^34^ with biopsy-proven MASLD over a mean follow-up of 8.4 years noted that the RES iron pattern was associated with an increased risk of fatal and nonfatal hepatic events. RES iron deposition was also associated with an increased risk of fatal and nonfatal cardiovascular events.^34^ A study using the NASH CRN database to study patients with various iron deposition patterns is currently underway.

A novel finding in the current study was that the presence of HC iron was associated with a lower likelihood of advanced fibrosis and milder MASH. Patients with HC iron had lower serum insulin and HOMA-IR levels, suggesting that the development of steatosis among these patients might be secondary to cytotoxic injury from HC iron, uncovering a distinct phenotype of MASLD among these patients.

In summary, this study in a well-characterized cohort of 2833 patients with MASH found that RES iron deposition was associated with higher disease severity, as evidenced by more severe histologic features and advanced fibrosis. In contrast, the presence of HC iron alone was associated with milder MASH and a lower frequency of advanced fibrosis. Considering that previous studies have noted that RES iron deposition was not associated with *HFE* mutations, further research using genome sequence data is needed to identify genetic variants that contribute to RES iron deposition. Finally, routine iron staining and assessment of the pattern of distribution of liver biopsies from patients with MASLD may provide valuable prognostic information.

## Supplementary Material

## References

[1] Rinella ME Lazarus JV Ratziu V Francque SM Sanyal AJ Kanwal F . A multisociety Delphi consensus statement on new fatty liver disease nomenclature. Hepatology. 2023;78:1966–1986. 37363821 10.1097/HEP.0000000000000520 PMC10653297

[2] Younossi ZM Golabi P Paik JM Henry A Van Dongen C Henry L . The global epidemiology of nonalcoholic fatty liver disease (NAFLD) and nonalcoholic steatohepatitis (NASH): A systematic review. Hepatology. 2023;77:1335–1347. 36626630 10.1097/HEP.0000000000000004 PMC10026948

[3] Targher G Byrne CD Tilg H . NAFLD and increased risk of cardiovascular disease: Clinical associations, pathophysiological mechanisms and pharmacological implications. Gut. 2020;69:1691–1705. 32321858 10.1136/gutjnl-2020-320622

[4] Noureddin N Copur-Dahi N Loomba R . Monitoring disease progression in metabolic dysfunction-associated steatotic liver disease. Aliment Pharmacol Ther. 2024;59(suppl 1):S41–S51. 38813822 10.1111/apt.17752 PMC11141723

[5] Sanyal AJ Van Natta ML Clark J Neuschwander-Tetri BA Diehl A Dasarathy S . Prospective study of outcomes in adults with nonalcoholic fatty liver disease. N Engl J Med. 2021;385:1559–1569. 34670043 10.1056/NEJMoa2029349 PMC8881985

[6] Letteron P Fromenty B Terris B Degott C Pessayre D . Acute and chronic hepatic steatosis lead to in vivo lipid peroxidation in mice. J Hepatol. 1996;24:200–208. 8907574 10.1016/s0168-8278(96)80030-4

[7] Berson A De Beco V Lettéron P Robin MA Moreau C Kahwaji JE . Steatohepatitis-inducing drugs cause mitochondrial dysfunction and lipid peroxidation in rat hepatocytes. Gastroenterology. 1998;114:764–774. 9516397 10.1016/s0016-5085(98)70590-6

[8] Rolo AP Teodoro JS Palmeira CM . Role of oxidative stress in the pathogenesis of nonalcoholic steatohepatitis. Free Radic Biol Med. 2012;52:59–69. 22064361 10.1016/j.freeradbiomed.2011.10.003

[9] Nelson JE Klintworth H Kowdley KV . Iron metabolism in nonalcoholic fatty liver disease. Curr Gastroenterol Rep. 2012;14:8–16. 22124850 10.1007/s11894-011-0234-4

[10] Nelson JE Wilson L Brunt EM Yeh MM Kleiner DE Unalp-Arida A . Relationship between the pattern of hepatic iron deposition and histological severity in nonalcoholic fatty liver disease. Hepatology. 2011;53:448–457. 21274866 10.1002/hep.24038 PMC3058264

[11] Valenti L Fracanzani AL Bugianesi E Dongiovanni P Galmozzi E Vanni E . HFE genotype, parenchymal iron accumulation, and liver fibrosis in patients with nonalcoholic fatty liver disease. Gastroenterology. 2010;138:905–912. 19931264 10.1053/j.gastro.2009.11.013

[12] Sun Z Pan X Tian A Surakka I Wang T Jiao X . Genetic variants in HFE are associated with non-alcoholic fatty liver disease in lean individuals. JHEP Rep. 2023;5:100744. 37235137 10.1016/j.jhepr.2023.100744 PMC10206181

[13] Valenti L Dongiovanni P Fracanzani AL Santorelli G Fatta E Bertelli C . Increased susceptibility to nonalcoholic fatty liver disease in heterozygotes for the mutation responsible for hereditary hemochromatosis. Dig Liver Dis. 2003;35:172–178. 12779071 10.1016/s1590-8658(03)00025-2

[14] Hernaez R Yeung E Clark JM Kowdley KV Brancati FL Kao WH . Hemochromatosis gene and nonalcoholic fatty liver disease: A systematic review and meta-analysis. J Hepatol. 2011;55:1079–1085. 21354231 10.1016/j.jhep.2011.02.013 PMC3611963

[15] Nelson JE Bhattacharya R Lindor KD Chalasani N Raaka S Heathcote JE . HFE C282Y mutations are associated with advanced hepatic fibrosis in Caucasians with nonalcoholic steatohepatitis. Hepatology. 2007;46:723–729. 17680648 10.1002/hep.21742

[16] Chitturi S Weltman M Farrell GC McDonald D Liddle C Samarasinghe D . HFE mutations, hepatic iron, and fibrosis: Ethnic-specific association of NASH with C282Y but not with fibrotic severity [published correction appears in *Hepatology* 2002 Nov;36(5):1307]. Hepatology. 2002;36:142–149. 12085358 10.1053/jhep.2002.33892

[17] Bugianesi E Manzini P D'Antico S Vanni E Longo F Leone N . Relative contribution of iron burden, HFE mutations, and insulin resistance to fibrosis in nonalcoholic fatty liver. Hepatology. 2004;39:179–187. 14752836 10.1002/hep.20023

[18] Yoneda M Nozaki Y Endo H Mawatari H Iida H Fujita K . Serum ferritin is a clinical biomarker in Japanese patients with nonalcoholic steatohepatitis (NASH) independent of HFE gene mutation. Dig Dis Sci. 2010;55:808–814. 19267193 10.1007/s10620-009-0771-y

[19] Nelson JE Brunt EM Kowdley KV Nonalcoholic Steatohepatitis Clinical Research Network . Lower serum hepcidin and greater parenchymal iron in nonalcoholic fatty liver disease patients with C282Y HFE mutations. Hepatology. 2012;56:1730–1740. 22611049 10.1002/hep.25856 PMC3462887

[20] George DK Goldwurm S MacDonald GA Cowley LL Walker NI Ward PJ . Increased hepatic iron concentration in nonalcoholic steatohepatitis is associated with increased fibrosis. Gastroenterology. 1998;114:311–318. 9453491 10.1016/s0016-5085(98)70482-2

[21] Mitsuyoshi H Yasui K Yamaguchi K Minami M Okanoue T Itoh Y . Pathogenic role of iron deposition in reticuloendothelial cells during the development of chronic hepatitis C. Int J Hepatol. 2013;2013:686420. 23653861 10.1155/2013/686420 PMC3638689

[22] Maliken BD Nelson JE Klintworth HM Beauchamp M Yeh MM Kowdley KV . Hepatic reticuloendothelial system cell iron deposition is associated with increased apoptosis in nonalcoholic fatty liver disease. Hepatology. 2013;57:1806–1813. 23325576 10.1002/hep.26238 PMC3637923

[23] Kanamori Y Tanaka M Itoh M Ochi K Ito A Hidaka I . Iron-rich Kupffer cells exhibit phenotypic changes during the development of liver fibrosis in NASH. iScience. 2021;24:102032. 33521599 10.1016/j.isci.2020.102032 PMC7820131

[24] Handa P Thomas S Morgan-Stevenson V Maliken BD Gochanour E Boukhar S . Iron alters macrophage polarization status and leads to steatohepatitis and fibrogenesis. J Leukoc Biol. 2019;105:1015–1026. 30835899 10.1002/JLB.3A0318-108R

[25] Kleiner DE Brunt EM Van Natta M Behling C Contos MJ Cummings OW . Design and validation of a histological scoring system for nonalcoholic fatty liver disease. Hepatology. 2005;41:1313–1321. 15915461 10.1002/hep.20701

[26] Rowe JW Wands JR Mezey E Waterbury LA Wright JR Tobin J . Familial hemochromatosis: Characteristics of the precirrhotic stage in a large kindred. Medicine (Baltimore). 1977;56:197–211. 870791

[27] Gao H Jin Z Bandyopadhyay G Wang G Zhang D Rocha KCE . Aberrant iron distribution via hepatocyte-stellate cell axis drives liver lipogenesis and fibrosis. Cell Metab. 2022;34:1201–1213.e5. 35921818 10.1016/j.cmet.2022.07.006 PMC9365100

[28] Dixon SJ Lemberg KM Lamprecht MR Skouta R Zaitsev EM Gleason CE . Ferroptosis: An iron-dependent form of nonapoptotic cell death. Cell. 2012;149:1060–1072. 22632970 10.1016/j.cell.2012.03.042 PMC3367386

[29] Tsurusaki S Tsuchiya Y Koumura T Nakasone M Sakamoto T Matsuoka M . Hepatic ferroptosis plays an important role as the trigger for initiating inflammation in nonalcoholic steatohepatitis. Cell Death Dis. 2019;10:449. 31209199 10.1038/s41419-019-1678-y PMC6579767

[30] Qi J Kim JW Zhou Z Lim CW Kim B . Ferroptosis affects the progression of nonalcoholic steatohepatitis via the modulation of lipid peroxidation–mediated cell death in mice. Am J Pathol. 2020;190:68–81. 31610178 10.1016/j.ajpath.2019.09.011

[31] Elumalai S Karunakaran U Moon JS Won KC . Ferroptosis signaling in pancreatic β-cells: Novel insights & therapeutic targeting. Int J Mol Sci. 2022;23:13679. 36430158 10.3390/ijms232213679 PMC9690757

[32] Kowdley KV Gochanour EM Sundaram V Shah RA Handa P . Hepcidin signaling in health and disease: Ironing out the details. Hepatol Commun. 2021;5:723–735. 34027264 10.1002/hep4.1717 PMC8122377

[33] Hoki T Miyanishi K Tanaka S Takada K Kawano Y Sakurada A . Increased duodenal iron absorption through up-regulation of divalent metal transporter 1 from enhancement of iron regulatory protein 1 activity in patients with nonalcoholic steatohepatitis. Hepatology. 2015;62:751–761. 25753988 10.1002/hep.27774

[34] Eder SK Feldman A Strebinger G Kemnitz J Zandanell S Niederseer D . Mesenchymal iron deposition is associated with adverse long-term outcome in non-alcoholic fatty liver disease. Liver Int. 2020;40:1872–1882. 32378295 10.1111/liv.14503 PMC7496452

