# Supplementary material for: Association between pattern of hepatic iron deposition and severity of MASH among patients enrolled in the NASH Clinical Research Network
Source: Hepatol Commun. 2025 Nov 20;9(12):e0847. doi: 10.1097/HC9.0000000000000847 (PMC12637332; doi:10.1097/HC9.0000000000000847)
Supplement: Supplementary file 1 [file hc9-9-e0847-s001.pdf]

Supplemental Table 1. Geographical distribution by iron positivity

**Clinical Center**	**Iron stain negative (N=1,667)**	**Iron stain positive (N=1,166)**	**P**
			<0.001
Cleveland, OH	281 (67%)	139 (33%)	
Durham, NC	239 (61%)	151 (39%)	
Indianapolis, IN	173 (56%)	135 (44%)	
Richmond, VA	250 (62%)	156 (38%)	
Saint Louis, MO	173 (60%)	113 (40%)	
San Diego, CA	185 (46%)	214 (54%)	
San Francisco, CA	157 (59%)	107 (41%)	
Seattle, WA	209 (58%)	151 (42%)	
Total	1,667 (59%)	1,166 (41%)	

For Peer Review
